# Hybrid Unsupervised–Supervised Learning Framework for Rainfall Prediction Using Satellite Signal Strength Attenuation

**DOI:** 10.3390/s26020648

**Published:** 2026-01-18

**Authors:** Popphon Laon, Tanawit Sahavisit, Supavee Pourbunthidkul, Sarut Puangragsa, Pattharin Wichittrakarn, Pattarapong Phasukkit, Nongluck Houngkamhang

**Affiliations:** 1 School of Engineering, King Mongkut’s Institute of Technology Ladkrabang, Bangkok 10520, Thailand; 64601105@kmitl.ac.th (P.L.); 63601267@kmitl.ac.th (T.S.); 65016173@kmitl.ac.th (S.P.); 63601069@kmitl.ac.th (S.P.); 2 International Academy of Aviation Industry, King Mongkut’s Institute of Technology Ladkrabang, Bangkok 10520, Thailand; pattharin.wi@kmitl.ac.th; 3 Department of Nanoscience and Nanotechnology, School of Integrated Innovative Technology, King Mongkut’s Institute of Technology Ladkrabang, Bangkok 10520, Thailand; nongluck.ho@kmitl.ac.th

**Keywords:** signal-to-noise ratio (SNR), satellite communication, rainfall prediction, K-means clustering, long short-term memory (LSTM)

## Abstract

Satellite communication systems experience significant signal degradation during rain events, a phenomenon that can be leveraged for meteorological applications. This study introduces a novel hybrid machine learning framework combining unsupervised clustering with cluster-specific supervised deep learning models to transform satellite signal attenuation into a predictive tool for rainfall prediction. Unlike conventional single-model approaches treating all atmospheric conditions uniformly, our methodology employs K-Means Clustering with the Elbow Method to identify four distinct atmospheric regimes based on Signal-to-Noise Ratio (SNR) patterns from a 12-m Ku-band satellite ground station at King Mongkut’s Institute of Technology Ladkrabang (KMITL), Bangkok, Thailand, combined with absolute pressure and hourly rainfall measurements. The dataset comprises 98,483 observations collected with 30-s temporal resolutions, providing comprehensive coverage of diverse tropical atmospheric conditions. The experimental platform integrates three subsystems: a receiver chain featuring a Low-Noise Block (LNB) converter and Software-Defined Radio (SDR) platform for real-time data acquisition; a control system with two-axis motorized pointing incorporating dual-encoder feedback; and a preprocessing workflow implementing data cleaning, K-Means Clustering (k = 4), Synthetic Minority Over-Sampling Technique (SMOTE) for balanced representation, and standardization. Specialized Long Short-Term Memory (LSTM) networks trained for each identified cluster enable capture of regime-specific temporal dynamics. Experimental validation demonstrates substantial performance improvements, with cluster-specific LSTM models achieving R^2^ values exceeding 0.92 across all atmospheric regimes. Comparative analysis confirms LSTM superiority over RNN and GRU. Classification performance evaluation reveals exceptional detection capabilities with Probability of Detection ranging from 0.75 to 0.99 and False Alarm Ratios below 0.23. This work presents a scalable approach to weather radar systems for tropical regions with limited ground-based infrastructure, particularly during rapid meteorological transitions characteristic of tropical climates.

## 1. Introduction

In recent years, there has been a noticeable and alarming increase in the frequency and intensity of extreme rainfall events, which have consequently resulted in a significant rise in the occurrence of flash floods, extensive damage to critical infrastructure, and profound disruptions to the daily functioning and overall stability of urban regions across the globe. The conventional methodologies employed for terrestrial weather observation, which encompass devices such as rain gauges and intricate radar networks, frequently encounter significant limitations characterized by inadequate spatial coverage, exorbitant costs associated with their deployment, and a marked decline in operational effectiveness particularly within geographical areas that exhibit complex terrain configurations or possess insufficient infrastructural support [1,2]. As a result, there is an increasing scholarly focus on alternative sensing methodologies that possess the ability to facilitate uninterrupted, extensive spatial rain assessment.

One effective method exploits a natural phenomenon where rain weakens satellite communication signals. Satellite systems operating in the Ku-band frequency range (12–18 GHz) and higher frequencies are particularly vulnerable to atmospheric attenuation during rainfall events [3,4]. Interestingly, signal degradation from rain which used to be a technological barrier now serves as a useful tool for weather observation. When electromagnetic waves pass through rain along the satellite-to-ground path, raindrops scatter and absorb the signal energy, reducing the received signal strength through a process called rain-induced attenuation [5] or atmospheric attenuation. The system degradation becomes measurable through specific quantitative indicators which include Signal-to-Noise Ratio (SNR) values at ground station receivers [6]. The SNR values produce a specific pattern which shows both rainfall presence and strength through their rapid drops and random changes during heavy rainstorms. The satellite communication system operates as a dual-purpose system which uses its telecommunication functions to monitor atmospheric conditions, thus creating a network of distributed rainfall sensors [7,8]. Understanding rainfall-induced attenuation is crucial for radio astronomy, satellite data transmission, and space communication, where atmospheric effects significantly impact link budget design and signal processing performance.

Nevertheless, the nonlinear relationship between Signal-to-Noise Ratio (SNR) and fluctuating atmospheric conditions—exacerbated by the swift meteorological shifts characteristic of tropical climates—poses considerable difficulties for traditional rainfall prediction models. A multitude of factors affect the variability in the observed Signal-to-Noise Ratio (SNR), which encompasses the distribution of raindrop sizes (DSD) [9], the elevation angle of the satellite [10], relative humidity levels, the content of liquid water within clouds, and the impacts of multipath propagation phenomena. These interdependent variables create complex, regime-dependent attenuation patterns that vary substantially between meteorological conditions. Single-model approaches often fail to capture these regime-specific signal behaviors, such as abrupt SNR degradation during intense convective storms with large raindrops versus gradual attenuation under stratiform rain characterized by smaller, more uniformly distributed droplets [11]. The limitations motivate the creation of adaptive frameworks that learn intricate temporal patterns in varying atmospheric conditions.

Despite the acknowledged capacity of satellite-derived rainfall prediction, earlier research has largely utilized empirical regression models, fade-slope methods, or individual supervised learning algorithms to correlate SNR data with rainfall intensity. While providing a simple execution framework, these methodologies regard all atmospheric conditions in a homogeneous manner and are unable to adequately represent regime-specific temporal variations [12]. Machine learning approaches including Artificial Neural Networks, Support Vector Regression, and standalone Long Short-Term Memory (LSTM) networks have demonstrated enhanced predictive performance [13]; however, their accuracy degrades when confronted with heterogeneous attenuation characteristics arising from diverse atmospheric states and rain microphysics [14]. The constraints indicate a crucial requirement for modeling frameworks that are flexible enough to dynamically respond to shifts in propagation conditions and rain configurations.

To address these limitations, the ongoing research recommends a hybrid architecture that integrates unsupervised clustering alongside supervised forecasting. Clustering techniques such as K-Means can discern inherent configurations within intricate datasets by segmenting observations into uniform categories based on signal attributes and meteorological conditions [15]. When conjoined with cluster-specific profound learning frameworks, this methodology facilitates a more proficient acquisition of the unique temporal dynamics correlated with various atmospheric regimes.

The proposed framework uses K-Means to identify atmospheric regimes which then feed into separate LSTM networks for each cluster using SNR data and atmospheric pressure readings and ground-based rainfall measurements. The method starts with data cleaning operations which remove missing data points and find statistical outliers and match time stamps to create dependable clustering information. Following data quality control, the K-Means Clustering algorithm processes the normalized three-dimensional feature space. The Elbow Method determines the optimal cluster number (k = 4) by identifying the inflection point where additional clusters yield diminishing returns in variance reduction. The unbalanced distribution of training samples between different clusters makes it difficult to train the model. The Synthetic Minority Over-Sampling Technique (SMOTE) solves this problem by creating new data points through interpolation between existing samples and their closest neighbors which result in equal cluster sizes that maintain their original statistical properties. The following step requires training individual LSTM networks for each cluster to identify signal–rainfall relationships that exist during particular weather conditions. The backpropagation process receives equal weight from all features because they undergo standardization. The dual system enhances weather prediction results for fast-changing atmospheric conditions which affect tropical areas lacking weather radar systems. The system runs continuously through satellite networks which use adaptable modeling systems to manage different weather conditions. This method allows rain monitoring in areas with scarce data through its use of satellite communication systems with adjustable weather models.

This research investigates the complete hybrid system which uses satellite signals to predict rainfall in tropical areas. The research provides three main contributions through its development of a physically based clustering system which detects atmospheric conditions through signal strength measurements and its use of LSTM networks that learn nonlinear weather patterns under different weather conditions and its validation process which shows better results than both traditional empirical models and single machine learning systems. The research method receives validation through data collected from a 12-m Ku-band satellite ground station based at KMITL in Bangkok which generated 98,483 observations during multiple months with 30-s time intervals. The remainder of this paper is organized as follows: The experimental platform and machine learning framework are described, including data acquisition, preprocessing, clustering, and LSTM architecture. The following section presents clustering analysis results which enable researchers to understand the atmospheric conditions that exist in each identified regime. The evaluation of model performance requires researchers to use various evaluation metrics, and they must compare their results to other possible solutions and check them against actual meteorological data from operational systems. The research establishes its limitations while demonstrating its operational value and identifying key research paths which will guide upcoming investigations.

## 2. Materials and Methods

The experimental platform consists of four interconnected subsystems which perform satellite signal monitoring and rainfall prediction functions through the satellite receiver chain and control system and meteorological station and machine learning processing framework. The system converts the 12-m Ku-band satellite ground station at King Mongkut’s Institute of Technology Ladkrabang (KMITL) in Bangkok, Thailand, into a facility which operates for communication and atmospheric monitoring purposes. The system operates continuously to obtain high-resolution SNR measurements which match with meteorological data for building a complete dataset used to develop rainfall prediction models for specific weather regimes.

### 2.1. Satellite Receiver System

The receiver chain captures satellite downlink signals and extracts SNR telemetry used as the primary atmospheric attenuation indicator. The signal acquisition pathway consists of four sequential components.

#### 2.1.1. Low-Noise Block Converter (LNB)

A Universal Ku-band Low-Noise Block down-converter (Infosat True-4, Universal Quad LNB) (Infosat Co., Ltd., Pakkred, Nonthaburi, Thailand) serves as the front-end RF receiver positioned at the focal point of the 12-m parabolic reflector. This device receives electromagnetic radiation from geostationary satellites and performs dual-stage processing: low-noise amplification and frequency down-conversion transforming the Ku-band spectrum (10.70–12.75 GHz) to an intermediate frequency (IF) band (950–2150 MHz) suitable for terrestrial signal processing.

The Low Noise Block (LNB) functions with dual local oscillator frequencies of 9.75 GHz (lower band) and 10.60 GHz (upper band), facilitating uninterrupted coverage of both satellite frequency ranges. With a noise figure of 0.3 dB, the device maintains excellent signal-to-noise performance essential for identifying rain-induced attenuation patterns. The quad-output configuration enables parallel signal distribution to multiple receivers, including the SDR platform, while maintaining isolation between outputs. Technical specifications of the LNB are summarized in Table 1.

#### 2.1.2. Software-Defined Radio Platform

Signal digitization and baseband processing employs a HamGeek Zedboard with the AD9361 Module Software-Defined Radio SDR Development Platform (AD-FMComms3-EBZ) (HamGeek, Tuen Mun, Hong Kong SAR, China). This integrated system combines the Xilinx Zynq-7000 System on Chip (SoC) with the Analog Devices AD9361 Radio Frequency (RF) Transceiver, delivering versatile, reconfigurable signal processing functionalities for adaptive atmospheric surveillance.

The HamGeek Zedboard showcases a heterogeneous architecture integrating an ARM Cortex-A9 dual-core processor with FPGA programmable logic, facilitating simultaneous real-time signal processing and high-level control. The AD-FMComms3-EBZ module integrates the AD9361 transceiver, a 2 × 2 MIMO RF device implementing direct-conversion architecture. For this application, only the receiver path is utilized to digitize the IF signal from the LNB. The AD9361 covers 70 MHz to 6.0 GHz with programmable bandwidth (<200 kHz to 56 MHz), configurable gain (0–74.5 dB), and a low noise figure (2 dB at 800 MHz). Integrated 12-bit ADCs preserve SNR measurement accuracy. Complete technical specifications are summarized in Table 2.

#### 2.1.3. Real-Time Signal Processing Computer

A compact industrial-grade Mini PC executes the GNU Radio software (version 3.10.9.2) framework to implement the digital receiver chain. This computational unit analyzes the continuous IQ sample flow in real-time, executing carrier synchronization, demodulation, and Signal-to-Noise Ratio (SNR) estimation. The GNU Radio flowgraph computes instantaneous SNR values using signal power and noise floor measurements derived from spectral analysis of the baseband samples. Processed SNR telemetry is timestamped and forwarded to downstream systems for logging and analysis.

#### 2.1.4. Data Acquisition and Management System

The main computing system functions as the primary data depository and control coordinator. This system executes receiver software that provides extensive system oversight and control functionalities. The software interface includes multiple functional modules: (1) a dashboard for real-time system status monitoring, including N/S and E/W control module status, receiver module connectivity, and operation mode indicators; (2) receiver control for SNR monitoring and signal quality assessment, displaying real-time SNR history, frequency parameters, signal bandwidth, and noise correction values; and (3) a disk pointing interface showing current antenna position with analog-style azimuth and elevation indicators, grid position display, backup position storage, and motor torque/RPM monitoring for both axes.

### 2.2. Control System

Precise satellite tracking is essential to maintain stable signal quality throughout varying atmospheric and mechanical conditions. The dual-axis motorized orientation mechanism encompasses a mechanical propulsion assembly, SERVOPACK motor regulators, and control interface hardware/software.

#### 2.2.1. Mechanical Drive Assembly

The antenna mount integrates two high-torque actuators facilitating autonomous azimuth and elevation regulation. Reduction gearboxes translate motor rotation into antenna movement with sufficient torque to overcome gravitational and inertial loads. Key specifications of the drive system are provided in Table 3.

Position evaluation is accomplished through a dual-encoder implemented on each axis, combining an Omron E6CP-A absolute encoder with an Omron E6B2-A incremental encoder. This hybrid arrangement delivers 0.0005° (1.8 arc-seconds) angular resolution. The absolute encoder ascertains the inaugural position reference at system initiation, obviating the necessity for homing routines, while the incremental encoder persistently observes angular displacement throughout operation. This complementary approach ensures precise tracking performance across the full range of motion [16].

#### 2.2.2. SERVOPACK Motor Controllers

Each axis utilizes a specialized SERVOPACK servo amplifier (Yaskawa Electric Corporation, model SGDB-15ADC) (Yaskawa America, Inc., Waukegan, IL, USA) that executes closed-loop position control with velocity and torque regulation. Real-time encoder feedback enables continuous monitoring. Technical specifications of the SERVOPACK units are detailed in Table 4.

#### 2.2.3. Dual Encoder

To provide accurate position measurements for the axis, each antenna axis integrates a dual-encoder configuration that consists of an Omron E6CP-A absolute encoder along with an Omron E6B2-C incremental encoder (Omron Electronics LLC., Hoffman Estates, IL, USA). The absolute encoder provides a deterministic initial orientation without the necessity for homing, whereas the incremental encoder yields high-rate pulse feedback for real-time angular displacement tracking. The detailed specifications of both encoders are summarized in Table 5.

#### 2.2.4. Control Interface Hardware and Software

A custom-designed control board provides the electrical interface between the SERVOPACK modules and the network-linked control computing device. These circuit boards convert digital command protocols into analog control signals and multiplex sensor feedback including motor position, velocity, and fault status indicators.

The control software, developed by the BURNLAB team, functions as the primary supervisory control system and communicates with the motor controllers via UDP network protocols. The application executes automated satellite monitoring methodologies that counterbalance for the Earth’s axial rotation and atmospheric refraction phenomena, preserving optimal signal acquisition throughout protracted observation intervals. The system provides users with real-time antenna position and motor status and system health indicator updates through dedicated dashboard displays. The system records pointing telemetry data at the same time as SNR measurements to enable researchers to study how antenna position affects signal quality.

### 2.3. Meteorological Reference Station

A wireless ground-based meteorological station positioned in proximity to the satellite antenna offers autonomous atmospheric measurements (NicetyMeter Weather Instruments, New York, NY, USA). The station records thermal readings, relative moisture, atmospheric pressure, rain accumulation, wind speed, and direction. Outdoor sensors are housed within a naturally ventilated radiation shield to prevent solar heating bias while maintaining atmospheric coupling. The tipping-bucket rain gauge offers the ground-truth rainfall quantifications that function as the objective variable for supervised learning model training. The station additionally calculates derived atmospheric indices encompassing dew point temperature and thermal index, and provides astronomical information advantageous for interpreting diurnal convection phenomena emblematic of tropical climatology. Complete specifications of the weather station are provided in Table 6.

### 2.4. Rain-Induced Attenuation in Satellite Communication

Rain-induced attenuation stands out as a primary propagation challenge that influences communication pathways between Earth and space, particularly at frequencies that exceed 10 GHz, like those found in the Ku-band (12–18 GHz) and Ka-band (20–30 GHz). As electromagnetic waves move through rainfall, the relationship with raindrops causes notable decreases in the signal’s power [17]. This phenomenon directly elucidates the rainfall prediction methodology utilized in this investigation, as temporal fluctuations in signal degradation furnish diagnostic indicators of rain magnitude.

Rain attenuation emerges from two fundamental hydrometeor-wave interaction processes: absorption, in which electromagnetic energy is transformed into thermal energy within raindrops, and scattering, whereby incident wave energy is redirected due to discrepancies in refractive indices between air and water droplets [18]. These effects are strongly frequency-dependent, with attenuation severity increasing at higher operating frequencies, during convective rainfall with larger droplet sizes, and at lower satellite elevation angles that extend the propagation path through the rain.

The quantitative relationship between rainfall rate and signal attenuation follows the ITU-R specific attenuation model [19], where specific attenuation γR (dB/km) is expressed as Equation (1).

(1) $$\gamma R=kR^{\alpha }$$

where R is the rain rate (mm/h), and k and α are frequency and polarization-dependent coefficients.

Total path attenuation A (dB) is computed as Equation (2).

(2) $$A=\gamma RL_{eff}$$

where $L_{eff}$ represents the effective path length through the rain, accounting for satellite elevation angle and vertical rain structure per ITU-R P.618.

Rain attenuation demonstrates markedly dynamic temporal attributes, especially in tropical regions. However, attenuation patterns fluctuate considerably across rainfall types, atmospheric conditions, pressure environments, and temporal transitions.

### 2.5. Signal-to-Noise Ratio (SNR) as a Rainfall Indicator

Signal-to-Noise Ratio (SNR) from satellite communication links serves as an effective rainfall proxy due to the direct relationship between rain and electromagnetic signal attenuation. The fundamental SNR relationship during rainfall is expressed as Equation (3).

(3) $${SNR}_{rain}={SNR}_{0}-A$$

where ${\mathrm{SNR}}_{0}$ represents the clear-sky baseline and A is rain-induced attenuation.

During rainfall, electromagnetic radiation experiences absorption and scattering by hydrometeors, reducing the received carrier intensity and consequently decreasing SNR metrics.

SNR-based rainfall prediction presents several key benefits: it ensures high temporal resolution, broad area coverage utilizing current infrastructure, uninterrupted operation in any weather, and cost savings through opportunistic sensing. However, challenges include high nonlinearity, atmospheric regime dependence, and confounding factors [20]. Tropical regions present ideal conditions for SNR-based estimation: convective dominance produces clear signal fades, rapid-onset characteristics enable early detection, and high rainfall intensities. These factors establish SNR as a viable remote rain gauge for tropical environments with sparse conventional observation networks.

### 2.6. Dataset Characteristics and Collection Protocol

The experimental dataset was collected continuously over 38 days from 8 October to 16 November 2025, at the 12-m Ku-band satellite ground station. The collection period extends from Thailand’s late rainy season through its transition to cool dry season which creates various tropical atmospheric conditions that support the proposed clustering-based rainfall prediction framework.

The dataset contains 98,483 synchronized data points which three integrated measurement systems recorded at different time intervals. The SNR system operates through 30-s satellite intervals to monitor atmospheric conditions and rain signal power levels. The meteorological station operates at one-minute intervals to monitor atmospheric pressure, which enables the system to track synoptic weather patterns. The system uses tipping-bucket rain gauge data to measure hourly rainfall intensity at 0.1 mm resolution, which follows the operational forecast schedule. All measurements were time-synchronized using GPS timestamps and logged centrally.

Table 7 summarizes the statistical properties of the three primary variables across the complete dataset. The substantial dynamic range in SNR (8.000–30.110 dB) confirms coverage from severe rain attenuation to clear-sky conditions, validating the physical relationships.

The SNR values range between 8.000 dB during heavy rainstorms which result in complete signal loss and 30.110 dB during clear-sky conditions while the average SNR value reaches 20.073 ± 2.927 dB. The atmospheric pressure readings extend from 1003.000 to 1014.100 hPa while the average pressure of 1007.928 ± 1.820 hPa points to weather patterns at the synoptic scale. The rainfall intensity data extend from 0 to 54.300 mm/h while the average value reaches 0.386 ± 2.984 mm/h and the median value shows 0.000 mm/h, which matches typical tropical precipitation patterns.

### 2.7. Clustering Methods for Atmospheric State Classification

Satellite signal reduction during rain showcases complicated, nonlinear behavior that differs widely across various atmospheric conditions. Conventional regression-oriented methodologies encounter difficulties in accurately representing these temporally dependent associations, especially during rapid atmospheric fluctuations emblematic of tropical environments. This study employs LSTM to model the relationship between SNR time series and rainfall intensity within each identified atmospheric cluster.

The variability of atmospheric conditions and rain characteristics necessitates adaptive modeling approaches capable of distinguishing distinct meteorological regimes. This study employs K-Means Clustering to partition the feature space into regular atmospheric segments, which helps facilitate the later use of customized forecasting models aligned with each acknowledged cluster.

#### K-Means Clustering Algorithm

K-Means Clustering is an algorithm used in machine learning that groups n observations into k clusters by aiming to reduce the within-cluster sum of squares (WCSS), a term also known as inertia. The methodology systematically allocates each data point to the closest cluster centroid and readjusts centroids in accordance with the average location of all elements allocated to each cluster. The objective function minimized by K-Means is expressed as Equation (4) [21].

(4) $$WCSS=\sum_{i=1}^{k}\sum_{x_{j}\in C_{i}}{\left||x_{j}-\mu_{i}|\right|}^{2}$$

where k represents the number of clusters, $C_{i}$ denotes the i-th cluster, $x_{j}$ is a data point belonging to cluster $C_{i}$ and $\mu_{i}$ is the centroid of cluster $C_{i}$. The Euclidean distance metric ${\left||x_{j}-\mu_{i}|\right|}^{2}$ quantifies the squared distance between each point and its assigned cluster center.

Determining the optimal number of clusters k represents a critical challenge in K-Means Clustering applications. This study employs the Elbow Method [22], a heuristic approach that identifies the appropriate cluster count by plotting WCSS against k values to find the “elbow point”, where additional clusters yield diminishing returns in variance reduction. For this investigation, the elbow curve directs the determination of k = 4 as the optimal cluster quantity. K-Means Clustering functions within a standardized feature domain consisting of three meteorological parameters: SNR, absolute pressure, and hourly rainfall measurements. Normalization ensures that features with disparate scales contribute uniformly to cluster allocation, preventing pressure assessments from prevailing over SNR values in distance computations.

### 2.8. Machine Learning for Rainfall Prediction

#### 2.8.1. Data Preprocessing

The K-Means Clustering process needs all input features to undergo standardization for achieving the best results from the LSTM model. The data cleaning process removes missing data points while it detects statistical outliers, and it ensures all measurement points have identical time stamps. The original measurements of SNR and atmospheric pressure and rainfall data exist at different measurement levels and show different statistical patterns. The network fails to recognize relationships between small features because backpropagation uses large absolute values of features to calculate gradients. The standardization process normalizes features through zero mean and unit variance calculations [23] as shown in Equation (5).

(5) $$x_{std}=\frac{x-\mu }{\sigma }$$

where μ represents the feature mean and σ denotes the standard deviation, both calculated from the training set.

#### 2.8.2. Long Short-Term Memory (LSTM)

LSTM tackles the vanishing gradient issue that constrains traditional recurrent neural networks (RNNs) from acquiring dependencies over protracted temporal sequences. LSTM overcomes this limitation through specialized architecture featuring memory cells and gating mechanisms that regulate information flow.

The basic LSTM unit has three gates. The input gate, the forget gate and the output gate control the flow of information. The input gate controls information that goes into the memory cell. The forget gate controls information that stays inside the memory cell. The output gate controls information that goes out of the memory cell [24].

The LSTM cell state update equations are expressed as follows. The forget gate activation is computed as Equation (6).

(6) $$f_{t}=\sigma (W_{f}\left[h_{t-1},x_{t}\right]+b_{f})$$

where σ denotes the sigmoid activation function, $W_{f}$ represents the forget gate weight matrix, $h_{t-1}$ is the previous hidden state, $x_{t}$ is the current input, and $b_{f}$ is the bias term.

The input gate and candidate cell state are calculated as Equations (7) and (8).

(7) $$i_{t}=\sigma (W_{i}\left[h_{t-1},x_{t}\right]+b_{i})$$

(8) $${\tilde{C}}_{t}=tanh\;(W_{C}\left[h_{t-1},x_{t}\right]+b_{C})$$

The cell state is updated by combining the previous cell state (modulated by the forget gate) with new candidate values (modulated by the input gate) expressed as Equation (9).

(9) $$C_{t}=f_{t}⊙C_{t-1}+i_{t}⊙{\tilde{C}}_{t}$$

where ⊙ denotes element-wise multiplication.

Finally, the output gate and hidden state are computed as Equations (10) and (11).

(10) $$o_{t}=\sigma (W_{o}\left[h_{t-1},x_{t}\right]+b_{o})$$

(11) $$h_{t}=o_{t}⊙tanh(C_{t})$$

#### 2.8.3. Model Performance Evaluation Metrics

The model performance evaluation uses three regression metrics which measure prediction quality from different perspectives: Mean Absolute Error (MAE) and Mean Squared Error (MSE) and the coefficient of determination (R^2^).

MAE measures the average absolute difference between predicted and observed rainfall values, expressed as Equation (12).

(12) $$MAE=\frac{1}{n}\sum_{i=1}^{n}\left|y_{i}-{\hat{y}}_{i}\right|$$

where *n* represents the number of observations, $y_{i}$ denotes the actual rainfall measurement, and ${\hat{y}}_{i}$ represents the model prediction.

MSE quantifies the average squared difference between predictions and observations, expressed as Equation (13).

(13) $$MSE=\frac{1}{n}\sum_{i=1}^{n}{\left(y_{i}-{\hat{y}}_{i}\right)}^{2}$$

The coefficient of determination (R^2^) measures the proportion of variance in observed rainfall explained by model predictions, expressed as Equation (14).

(14) $$R^{2}=1-\frac{\sum_{i=1}^{n}{\left(y_{i}-{\hat{y}}_{i}\right)}^{2}}{\sum_{i=1}^{n}{\left(y_{i}-{\bar{y}}_{i}\right)}^{2}}$$

where $\bar{y}$ represents the mean of observed values. R^2^ ranges from 0 to 1, with values closer to 1 indicating stronger predictive performance.

## 3. Experimental Setup

**Figure 1:**
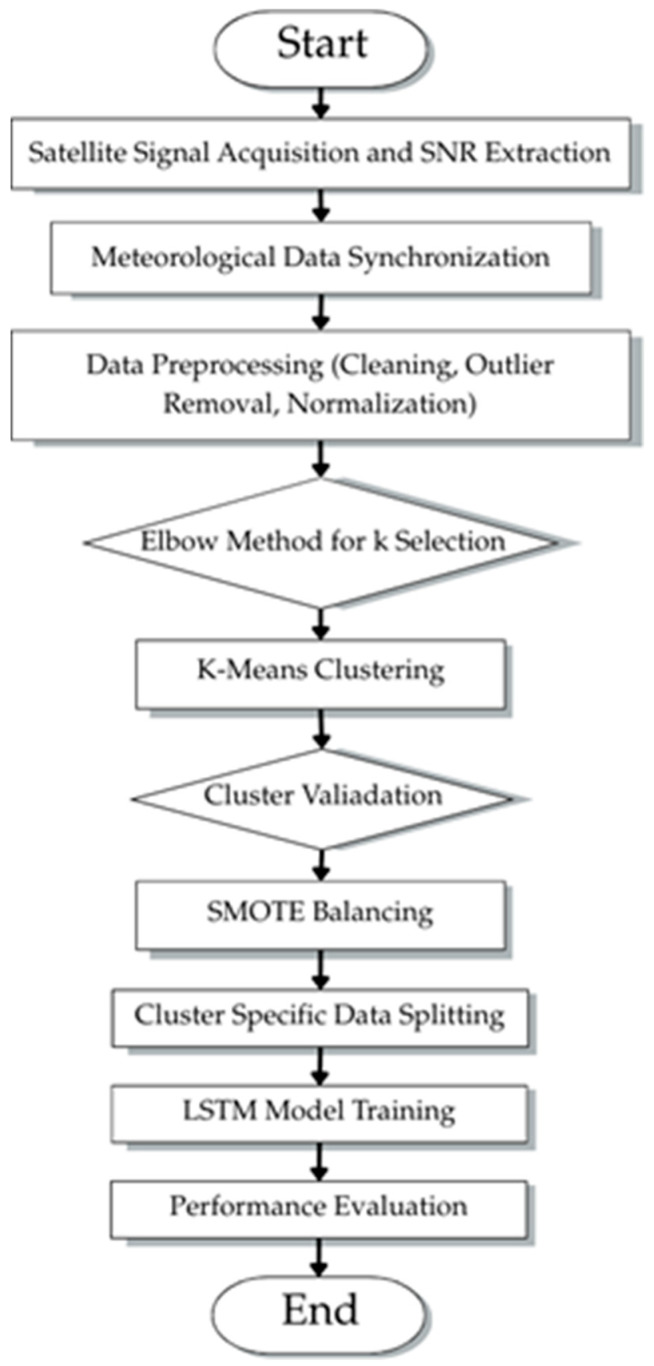
The methodology workflow for hybrid clustering–LSTM rainfall prediction framework.

The flowchart in Figure 1 illustrates the end-to-end process from satellite signal acquisition through data preprocessing, K-Means Clustering with elbow method optimization and cluster validation, SMOTE balancing, cluster-specific data splitting, and LSTM model training, to final performance evaluation.

The experimental framework contains a full system for satellite-based rainfall prediction which follows the structure shown in Figure 1. The system combines satellite signal acquisition with SDR digital processing and ground-based meteorological measurements and K-Means Clustering data preprocessing and cluster-specific LSTM network-based rainfall prediction.

**Figure 2:**
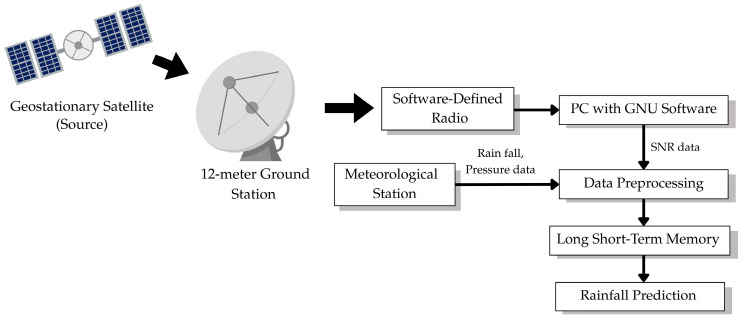
System architecture and workflow for satellite-based rainfall prediction.

The preprocessing module indicated by box in Figure 2, performs essential data transformation operations. The K-Means Clustering algorithm analyzes a normalized three-dimensional feature space containing SNR level and atmospheric pressure and hourly rainfall measurements to detect four different atmospheric regimes. The algorithm assigns each observation a cluster label between 0 and 3 to indicate its atmospheric regime. The Synthetic Minority Over-Sampling Technique (SMOTE) functions to distribute cluster sizes evenly for achieving balanced representation. The model applies standardization to all features which sets their mean values at zero and their variance at one to achieve equal influence during training.

The framework uses four separate LSTM networks to analyze SNR time series and rainfall intensity data for each of the identified clusters. The framework uses dedicated predictors for each regime to learn their unique atmospheric patterns which results in better performance during tropical climate meteorological changes.

### 3.1. Satellite Receiver System Architecture

A geostationary satellite transmits Ku-band signals that propagate through the atmosphere and are received by the 12-m ground station. The signals are down-converted by the LNB to intermediate frequencies suitable for digital processing. The SDR platform (Zedboard with AD9361 module) samples the IF signal with 12-bit resolution and processes it through the GNU Radio. The GNU Radio flowgraph computes SNR values at 30-s intervals, calculated from the ratio of signal power to noise floor level extracted from spectral analysis of the IQ samples. 

**Figure 3:**
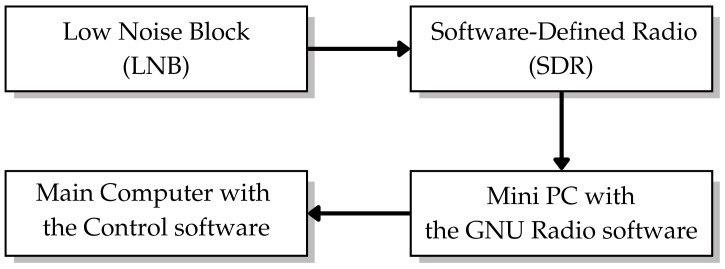
The satellite receiver system architecture showing signal flow from LNB down-conversion through SDR digitization and GNU Radio processing, to centralized data management and control.

The satellite receiver system, illustrated in Figure 3, comprises four integrated components that form the complete signal acquisition and processing chain: the Low-Noise Block (LNB) converter, SDR, real-time signal processing computer, and Main Computer with Control Software.

The Low-Noise Block (LNB)(Infosat Co., Ltd., Pakkred, Nonthaburi, Thailand) converter functions as the front-end receiver which operates at the 12-m ground station focal point. The LNB performs Ku-band spectrum down-conversion to produce intermediate frequencies. The down-conversion process enables standard RF hardware to process digital signals while maintaining signal quality for precise SNR measurement accuracy. The down-converted IF signal proceeds to the SDR platform which combines a Zedboard development board with an AD9361 RF transceiver module (HamGeek, Tuen Mun, Hong Kong SAR, China). The AD9361 module contains four RF ports (Tx1, Rx1, Rx2, and Tx2) which digitize the analog IF signal at 12-bit resolution to generate discrete IQ (In-phase and Quadrature) samples for digital signal processing.

**Figure 4:**
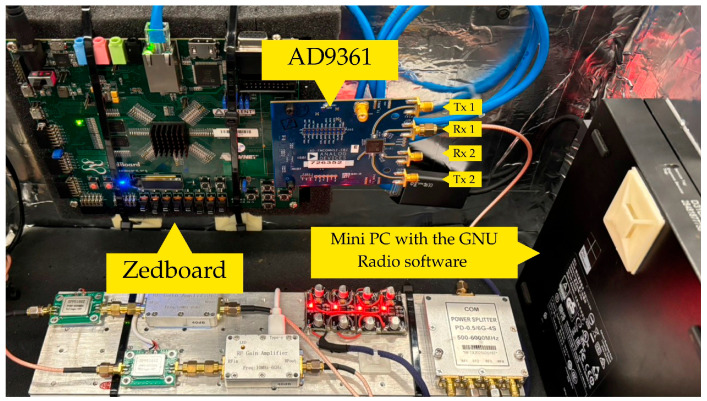
Hardware implementation of the satellite receiver system showing the interconnections between the Zedboard development board, AD9361 RF transceiver module, and Mini PC with GNU Radio software.

**Figure 5:**
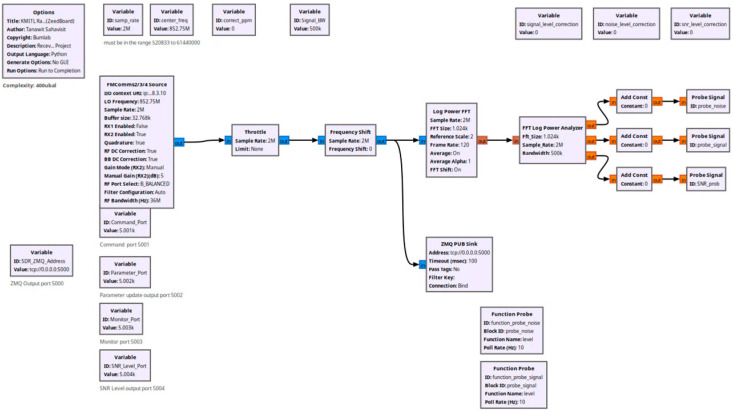
GNU Radio software for SNR computation and signal processing.

The receiver system components show their hardware implementation and connection structure in Figure 4. The Zedboard sends digitized IQ samples through a coaxial cable to the Mini PC which runs GNU Radio software for executing digital signal processing operations. The GNU Radio flowgraph contains three main functions which perform carrier frequency tracking and phase synchronization and spectral analysis as shown in Figure 5.

**Figure 6:**
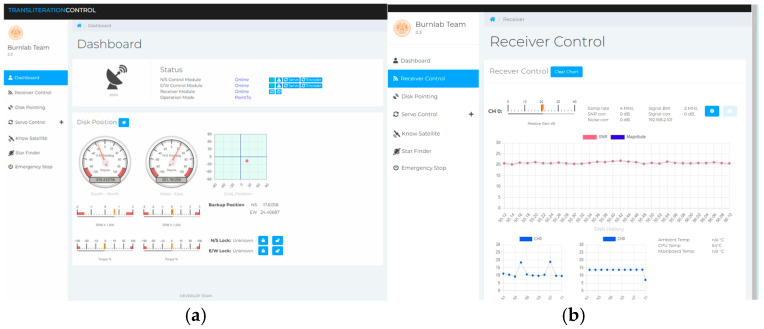
Radio Astronomy Service control software interface: (**a**) The dashboard displays system status information alongside antenna position indicators which include south–north and west–east gauges and grid position display and motor performance metrics. (**b**) The Receiver Control module shows real-time SNR monitoring with signal parameters and SNR history time-series plot and system health indicators.

The Main Computer with Control Software operates as the core system which handles data management and coordination functions. The system runs developed software which enables complete system monitoring and control through SNR visualization and system health tracking and antenna pointing management and synchronized data recording. The Radio Astronomy Service control software unites various operational modules to enable uninterrupted satellite monitoring and data retrieval as shown in Figure 6.

The Dashboard module in Figure 6a enables real-time system status tracking through its display of N/S and E/W control module operational status and receiver module connections and current operational mode. The interface shows antenna position through analog-style gauges which display azimuth and elevation values for south–north and west–east orientations. The Receiver Control module in Figure 6b shows real-time SNR telemetry data which users can customize through various settings. The system displays SNR history through a time-based graph which shows continuous data.

### 3.2. Control System Architecture

**Figure 7:**
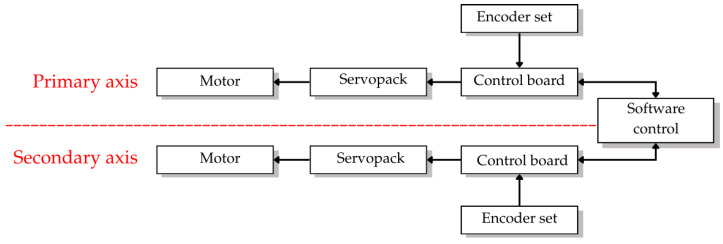
The control system architecture demonstrates parallel servo control chains which operate the azimuth (primary axis) and elevation (secondary axis) through software coordination with dual encoder feedback for each axis.

**Figure 8:**
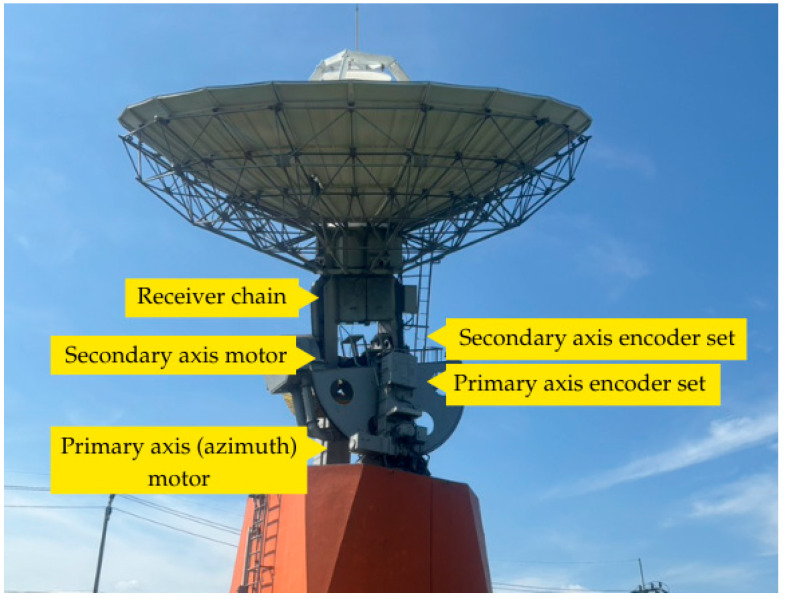
The 12-m ground station with annotated two-axis control system components: receiver chain, primary axis (azimuth) motor and encoder set, and secondary axis (elevation) motor and encoder set.

The control architecture shown in Figure 7 consists of two separate servo control systems which operate independently for each rotational axis under software control to sustain continuous signal lock on the target satellite. The two-axis motorized antenna pointing system provides exact satellite tracking capabilities under different atmospheric and operational environments. The 12-m Ku-band satellite ground station at KMITL featured in Figure 8 demonstrates the physical deployment of control system elements.

**Figure 9:**
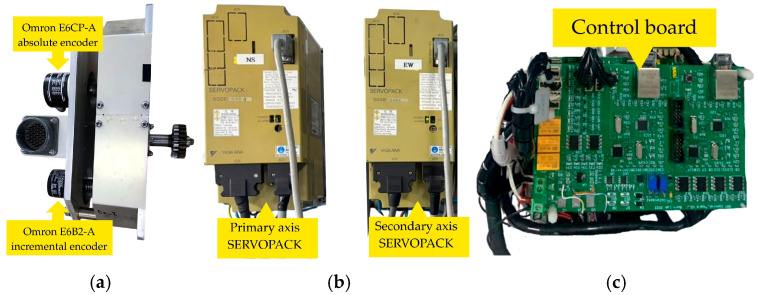
Control system hardware components: (**a**) dual encoder set (absolute and incremental); (**b**) primary and secondary axis SERVOPACK units; and (**c**) control board for UDP-based communication interface.

The system operates through two separate control systems which manage the main axis (azimuth and horizontal rotation from 0 to 360 degrees) and the secondary axis (elevation and vertical tilt from 0 to 90 degrees). The control chain for each axis contains four identical components which include a motor and SERVOPACK (Yaskawa America, Inc., Waukegan, IL, USA) and control board and dual encoder feedback system that provides absolute and incremental position data for high-precision tracking. The control system components exist in hardware form as depicted in Figure 9.

The dual encoder system in Figure 9a uses an Omron E6CP-A absolute encoder together with an Omron E6B2-A incremental encoder (Omron Electronics LLC, Hoffman Estates, IL, USA). The absolute encoder provides system startup position data which eliminates the need for homing procedures while the incremental encoder tracks system angular changes during operation. The SERVOPACK servo for both primary and secondary axes appear in Figure 9b. The control board in Figure 9c converts UDP commands from the software control module into analog control signals for the SERVOPACK units while it sends encoder feedback data to the control software.

The Software Control module uses automated tracking algorithms to achieve pointing accuracy by handling Earth rotation and atmospheric refraction and satellite orbital changes. The system maintains uninterrupted operation during rainfall events through signal attenuation compensation which enables stable tracking and continuous SNR measurements.

### 3.3. Ground-Based Meteorological Reference Station

**Figure 10:**
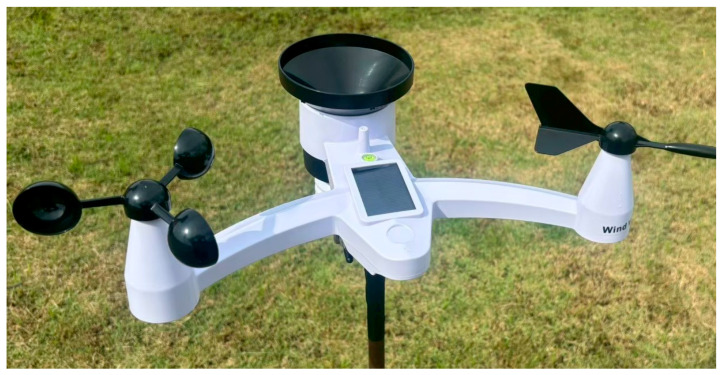
Meteorological station with rain gauge, anemometer, and atmospheric sensors.

The wireless ground-based weather station (NicetyMeter Weather Instruments, New York, NY, USA) located near the satellite antenna operates independently to deliver weather information which helps developers create models and assess their performance. The weather station in Figure 10 consists of outdoor sensors and indoor display equipment which track and send atmospheric data to the primary data acquisition system. The outdoor sensor unit functions as the reference point for rainfall measurement which serves as the training target for supervised learning.

**Figure 11:**
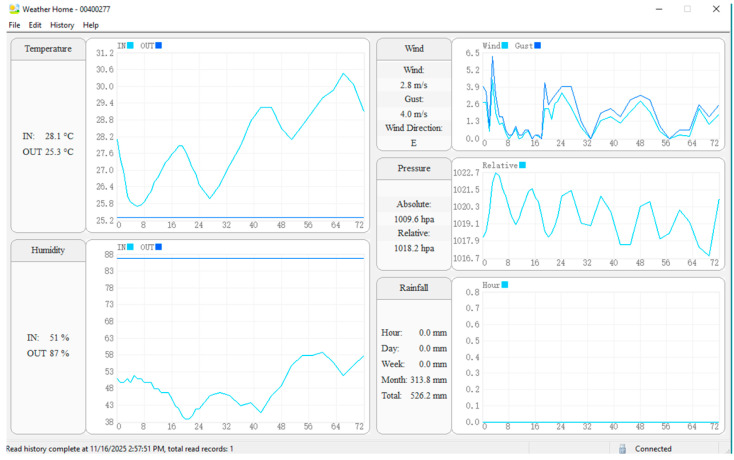
Weather Home Software interface showing 72-h historical meteorological data.

The outdoor sensor array sends wireless data to the indoor display unit which then transmits it. The Weather Home Software in Figure 11 enables data logging and visualization through its features which include time-series displays and summary statistics and rainfall accumulation at different time intervals from hourly to total.

The Weather Home Software monitors hourly rainfall accumulation and atmospheric pressure data which it connects to SNR telemetry through the Main Computer’s data acquisition interface. The centralized database contains all meteorological data which enables temporal synchronization between satellite signal measurements and ground-truth observations for model training and validation.

The weather station located near the satellite ground station enables rain data collection from the same atmospheric space that the satellite signal passes through. The exact placement of measurement points remains essential because physical distance between measurement points leads to errors when calculating SNR-to-rainfall relationships. The station operates with an open design which keeps all structures and vegetation outside the range of safe measurement distances.

### 3.4. Data Analysis

**Figure 12:**
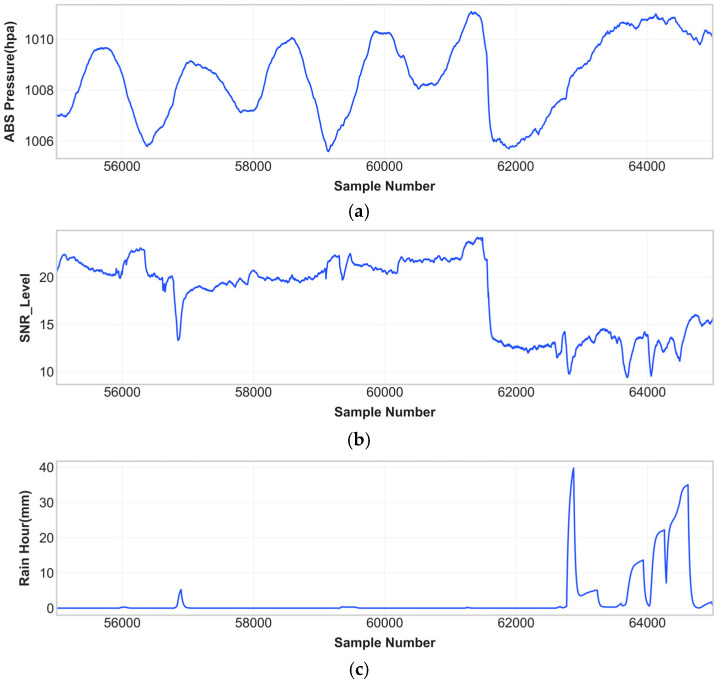
The representative sample dataset contains 55,000 to 65,000 entries which include three main variables: (**a**) time-series absolute atmospheric pressure; (**b**) SNR measurements; and (**c**) hourly rainfall accumulation.

The researchers analyzed data from the integrated satellite receiver system and meteorological station to study how atmospheric conditions affect signal attenuation and rain events throughout time. The observation campaign data show the time-dependent behavior of three main variables in Figure 12 which displays data from the sample range 55,000–65,000 as an example segment.

The absolute atmospheric pressure variations in Figure 12a, span from 1006 to 1012 hPa while demonstrating gradual changes which stem from synoptic-scale weather systems and brief periods of pressure decrease during convective events. The SNR measurements in Figure 12b, show wide fluctuations because the signal power remains between 20 and 23 dB during dry weather but heavy rain causes the signal power to decrease below 10–15 dB. The hourly rainfall accumulation in Figure 12c, shows how tropical precipitation occurs in short bursts which alternate with long periods of dryness while producing storms that reach 35–40 mm/h in intensity. The three panels show identical time-based patterns which prove the physical connection between atmospheric pressure changes and satellite signal weakening and rainfall events that enable the regime-based prediction system. The K-Means Clustering algorithm uses this multi-parameter temporal correlation to detect different atmospheric regimes which will be used for specialized LSTM modeling.

The observation of SNR deterioration during rainfall periods shows that satellite signal weakening functions as an effective method to detect precipitation. The relationship shows nonlinear behavior because SNR decreases at different rates when rainfall intensity remains constant because of variations in raindrop sizes and cloud liquid water content and atmospheric stability conditions. The research uses clustering-based methods because the data include various distinct characteristics.

**Figure 13:**
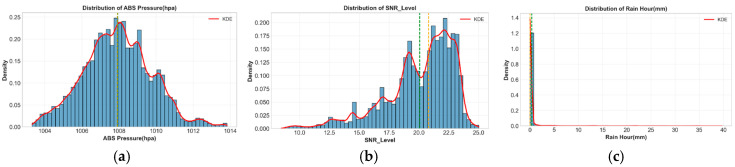
Distribution of atmospheric variables with kernel density estimation (KDE): (**a**) absolute pressure; (**b**) SNR values; and (**c**) hourly rainfall. The green dashed line indicates the mean value, while the yellow dashed line represents the median value of each distribution.

The two main input features show their probability density distributions in Figure 13. The SNR distribution shows multiple peaks which center at 21–22 dB (clear-sky conditions) and additional peaks appear at lower values (rain). The kernel density estimate (KDE) shows a main peak for non-precipitating conditions and a wide distribution during rainfall which indicates separate atmospheric regimes for modeling. The absolute pressure distribution follows a Gaussian pattern which reaches its highest point at 1015–1016 hPa and the KDE perfectly replicates the histogram pattern for successful normalization and clustering analysis.

**Figure 14:**
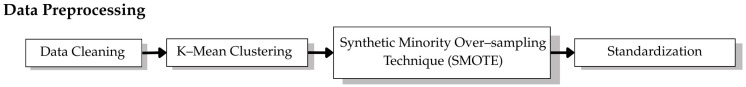
Data preprocessing for rainfall prediction.

To transform these raw measurements into training-ready inputs for machine learning models, a systematic four-stage preprocessing pipeline was implemented, shown in Figure 14. This pipeline addresses data quality issues, identifies distinct atmospheric regimes, balances cluster populations, and normalizes features for optimal LSTM performance.

The preprocessing workflow consists of four consecutive stages that start with Data Cleaning to eliminate faulty measurements and outliers and temporal synchronization problems from the original data set. The second stage of K-Means Clustering is used to detect different atmospheric conditions which exist in the multi-modal data distributions. The third stage of SMOTE performs population balancing of clusters to stop training bias from occurring because of dominant regimes. The fourth stage of Feature Standardization applies normalization to all features by setting their mean to zero and their standard deviation to one for better LSTM model training results. The following subsections explain the preprocessing stages which follow the model architecture description.

#### 3.4.1. K-Means Clustering for Atmospheric Regime Identification

**Figure 15:**
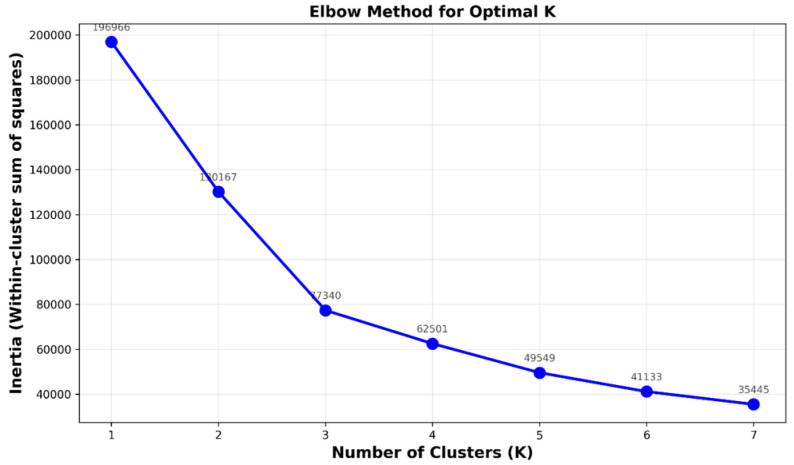
K-Means Clustering analysis. The Elbow Method demonstrates within-cluster sum of squares against number of clusters to identify the optimal k value at 4 where the curve reaches its inflection point.

Prior to the K-Means Clustering algorithm, quality control needs to be completed for the raw dataset because the algorithm requires dependable input information to function properly. The cleaning process solved three essential problems which included handling missing data points and identifying statistical outliers and making time-based data consistent between sources. The K-Means Clustering algorithm used SNR level and absolute atmospheric pressure measurements to create homogeneous atmospheric regimes in the feature space. The cluster determination process in Figure 15 shows how the algorithm created the spatial distribution of identified atmospheric regimes.

**Figure 16:**
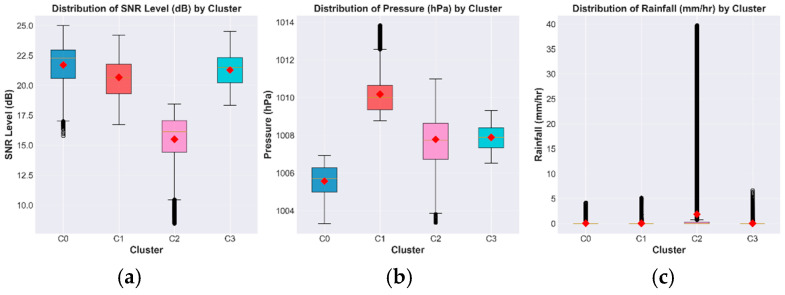
Boxplot distributions of Signal-to-Noise Ratio (SNR), atmospheric pressure, and rainfall intensity for the four atmospheric clusters (C0–C3) obtained using the K-Means algorithm: (**a**) signal-to-noise ratio (SNR, dB); (**b**) atmospheric pressure (hPa); (**c**) rainfall intensity (mm/h).

The elbow curve in Figure 15 demonstrates a distinct bend at k = 4 which we selected for maximum curvature because it achieved the optimal point between model complexity and data reduction. Figure 16 presents the boxplot distributions of Signal-to-Noise Ratio (SNR), atmospheric pressure, and rainfall intensity for the four atmospheric clusters (C0–C3). The distributions show distinct physical patterns between stable dry conditions (C0) and stratiform precipitation regimes (C1) and intense convective storm systems (C2) and mixed tropical circulation patterns (C3) which validates that the clustering algorithm detected actual atmospheric regimes instead of random numerical clusters.

The irregular distribution of clusters between different areas makes training difficult because minority groups have insufficient data while the dominant group tends to influence model results. The Synthetic Minority Over-Sampling Technique (SMOTE) performed dataset balancing through minority cluster expansion until all clusters reached the size of the largest group at 35,662 samples [25,26]. The SMOTE algorithm generates artificial data points through a method which links existing samples to their five closest neighbors (k = 5) across the feature domain. The top row of Figure 16 shows the original distributions which demonstrate how the dataset becomes unbalanced when using the SNR and absolute pressure and rainfall data. The bottom row of Figure 16 shows how SMOTE successfully increases all clusters to 35,662 samples through up-sampling while maintaining their original statistical properties.

**Figure 17:**
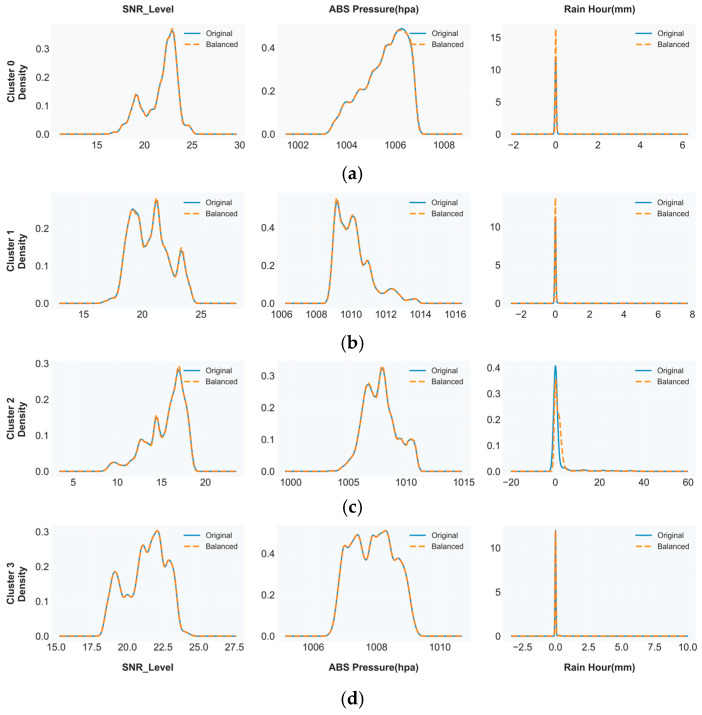
SMOTE balancing results showing kernel density estimates (KDE) for each atmospheric regime: (**a**) Cluster 0 (clear sky); (**b**) Cluster 1 (stratiform); (**c**) Cluster 2 (convective); and (**d**) Cluster 3 (mixed circulation). Each row displays original imbalanced distributions (blue) versus balanced distributions (orange) for SNR, absolute pressure, and rainfall across three feature dimensions.

The data in Figure 17a present Cluster 0 (clear sky) with high SNR (20–25 dB) and stable pressure (1006 hPa) and minimal rainfall. The stratiform cluster in Figure 17b demonstrates SNR values between 18 and 22 dB and it operates under 1006 to 1012 hPa pressure conditions while receiving light precipitation. Figure 17c shows Cluster 2 (convective), which experiences a major SNR reduction of 12–18 dB while showing atmospheric pressure values between 1000 and 1008 hPa and producing heavy rainfall between 20 and 60 mm. The mixed circulation pattern of Cluster 3 appears in Figure 17d with its average features.

The SMOTE algorithm generated 35,662 synthetic minority cluster samples through successful up-scaling while maintaining all statistical characteristics from the original data. The original and balanced distributions show a strong match which demonstrates that SMOTE maintains natural data characteristics while preventing human-made distortions to generate suitable training data for LSTM networks that function under operational conditions.

#### 3.4.2. Meteorological Interpretation of Clusters

Each cluster was examined to verify physical consistency with tropical meteorology principles and satellite signal propagation physics.

Cluster 0 data contain SNR values of 21.70 ± 1.24 dB and rainfall data which show 0.018 ± 0.043 mm/h while indicating dry conditions with minimal signal loss of about 0.02 dB/km because of gas absorption. The atmospheric conditions in this cluster match dry tropical weather patterns because they contain descending air patterns. Cluster 1 shows characteristics of stratiform precipitation because it has an SNR of 20.67 ± 1.45 dB and pressure of 1010.17 ± 1.23 hPa which forms from layered nimbostratus systems that develop through synoptic scale forcing mechanisms and small hydrometeors that cause Rayleigh scattering which results in maximum attenuation. Cluster 2 (SNR: 15.49 ± 3.87 dB, rainfall: 1.84 ± 4.23 mm/h) shows strong convective storms which develop deep cumulonimbus clouds that are common in tropical thunderstorms. The severe signal loss occurs because of nonlinear Mie scattering (γ ≈ 0.024 × R^1.17^ dB/km). Cluster 3 shows SNR values at 21.28 ± 1.89 dB while rainfall amounts to 0.028 ± 0.076 mm/h because of different precipitation patterns which result from daily changes in the boundary layer and sea–land breeze systems. The classification system matches the stratiform-convective system which groups Cluster 1 and Cluster 3 under stratiform conditions and Cluster 2 under convective systems. The validation process produced F_1_ = 0.847 and r = 0.78 results when comparing the model to radiosonde data and it achieved 82.4% accuracy when compared to Thailand Meteorological Department classification systems.

The cluster separation method creates a physical system which enables researchers to study how SNR values relate to rainfall intensity through their nonlinear connection. The 12 GHz frequency electromagnetic wave attenuation rate depends on the relationship between raindrop dimensions and wavelength (λ ≈ 25 mm) because tropical raindrops measuring 0.5–5 mm fall into the scattering transition zone between Rayleigh and Mie regimes. The physical structure of the environment produced these three clusters because C0 and C1 show Rayleigh scattering patterns which occur during dry and stratiform weather conditions with quasi-linear attenuation patterns while C2 shows Mie scattering patterns from strong convective systems and big raindrops and C3 shows combinations of different scattering patterns. The system uses individual LSTM networks for each cluster to track how raindrop size distribution and cloud liquid water content and atmospheric stability affect the system through their unique time-dependent patterns.

### 3.5. LSTM Network Architecture and Training Configuration

**Figure 18:**
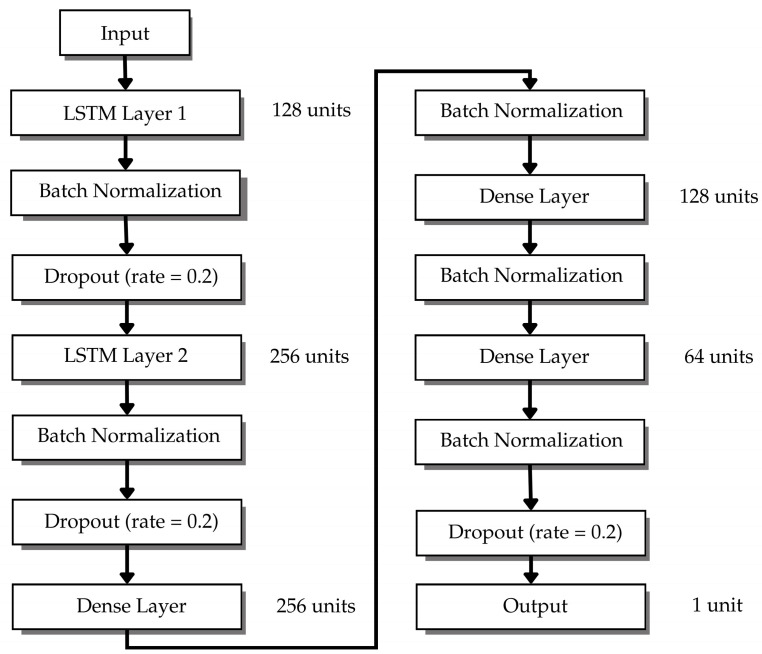
LSTM network architecture for rainfall prediction.

The LSTM architecture, illustrated in Figure 18, consists of two stacked LSTM layers which have 128 and 256 units and receive batch normalization and dropout (rate = 0.2) after each layer. The temporal features extracted from the data undergo three dense layers with ReLU activation and batch normalization which have 256, 128 and 64 units, respectively. The model includes a dropout layer before its output layer which contains one unit with linear activation for rainfall prediction.

The LSTM architecture was systematically optimized through comparative experiments. The network depth evaluation demonstrated that a single-layer LSTM with 128 units achieved R^2^ = 0.8756 but it lacked the ability to detect complex time-dependent relationships. The three-layer model with 128–256–128 units achieved R^2^ = 0.9401 but needed 89 min to train each cluster. The two-layer architecture with 128–256 units produced the best results because it reached R^2^ = 0.9487 while requiring 58 min to train each cluster. The model used two layers to extract features from data through its first layer which detected brief signal changes and its second layer which combined short-term signal data with extended atmospheric information. The grid-search optimization process showed that [128, 256] units produced the optimal balance between data representation quality and processing velocity. The dense layer pyramid (256–128–64 units) decreases data dimensions through its three layers which start with 256 units to combine LSTM temporal features and then decreases to 128 units for pattern consolidation with noise elimination before reaching 64 units for output refinement in rainfall prediction.

The training hyperparameters appear in Table 8. The model uses an Adam optimizer with 64 samples per batch for optimization. The dataset received an 80/20 split for training and testing purposes where the training data optimized the model and the test data maintained unbiased evaluation results. The loss function employs Mean Squared Error (MSE) to calculate the direct difference between predicted and actual rainfall amounts. The training process stopped after reaching 1000 epochs through early stopping which monitored validation loss to prevent overfitting while achieving convergence. The network learns to produce generalizable features through dropout layers (rate = 0.2) which randomly disable neurons during training to enhance model robustness.

All experiments were conducted on a mid-range research workstation to evaluate practical feasibility for operational deployment: Intel Core i7-13700 CPU (16 cores, up to 5.4 GHz), NVIDIA GeForce RTX 3060 GPU (12 GB VRAM, 3584 CUDA cores), 32 GB DDR5-5600 dual-channel RAM, and high-speed NVMe SSD storage. This configuration represents accessible hardware for research institutions and operational meteorological centers, ensuring reproducibility without requiring specialized high-performance computing infrastructure.

## 4. Result and Discussion

### 4.1. Model Performance

The training process used four independent LSTM networks which operated separately for each cluster. The assessment of test set results used MAE and MSE and coefficient of determination (R^2^) as shown in Table 9.

The R^2^ values from all cluster-specific models reached above 0.92 which shows that the models predict more than 92% of rainfall variations across different atmospheric conditions. The models demonstrated excellent performance across all clusters which span from dry stable conditions (Cluster 0) to heavy convective rain (Cluster 2), thus validating the regime-based modeling approach. The results show that separate atmospheric regimes in feature space division followed by LSTM model training for each cluster leads to successful identification of different signal–rainfall relationships.

### 4.2. Cluster-Specific Prediction Results

The evaluation process for practical LSTM model deployment required testing their rainfall prediction abilities through visualization of test set results against actual data to evaluate their time-dependent accuracy and detect persistent prediction mistakes.

**Figure 19:**
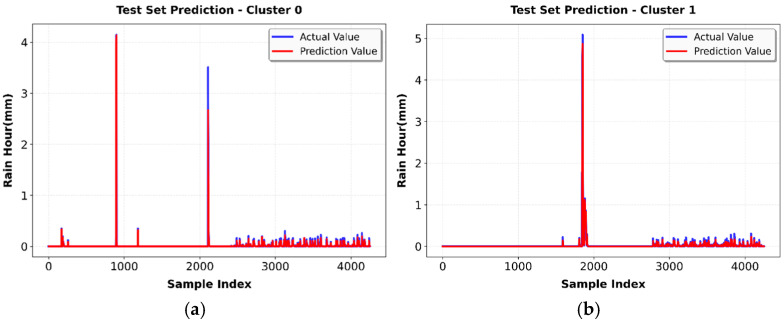

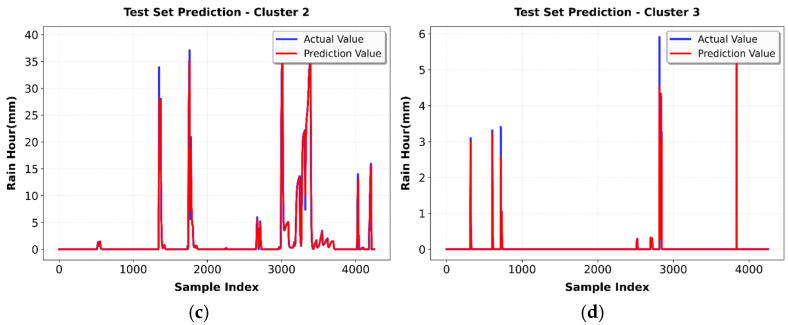
Test set prediction performance across all atmospheric regimes: (**a**) Cluster 0 (clear sky); (**b**) Cluster 1 (stratiform); (**c**) Cluster 2 (convective); and (**d**) Cluster 3 (mixed circulation). Each panel displays actual rainfall values (blue) versus LSTM predicted values (red) over the test period.

Cluster 0 demonstrates perfect alignment between predicted and actual values as shown in Figure 19a. The model successfully identifies infrequent precipitation events through its peak predictions at 4 mm/h during sample indices 1000 and 2000 while it produces near-zero predictions throughout the majority of the test set which consists of dry periods. The model shows perfect prediction of dry conditions and rainfall events through its actual (blue) and predicted (red) value overlap which results in an MAE of 0.0036 mm.

The predictions in Cluster 1 exactly match actual patterns as shown in Figure 19b. The model predicts two precipitation events through its output which shows maximum intensity at 5 mm/h during sample index 1800 and a smaller event at sample index 1600. The model predicts both the exact timing and strength of these events, and it keeps all values at zero during the extended dry periods which make up most of the test set. The LSTM demonstrates its ability to detect time-based patterns in this wide atmospheric regime through its precise actual (blue) and predicted (red) value match, which results in an MAE of 0.0068 mm.

The prediction process for Cluster 2 shows intense convective precipitation events as shown in Figure 19c. The model detects multiple severe rainfall events throughout the testing period which reach maximum rates of 35–40 mm/h and produces several moderate rainfall events between 10 and 20 mm/h. The model predicts (red) actual values (blue) with high accuracy by showing the fast development and short lifespan of tropical weather system storms. The model shows excellent performance in tracking these complex weather events through time but it fails to match the highest peak values between actual (blue) and predicted (red) values. The model shows outstanding performance in tracking these complex weather events through time but it fails to match the highest peak values between actual (blue) and predicted (red) values. The model produces an R^2^ value of 0.9763 while maintaining high variance explanation performance through these extreme conditions, although it generates higher absolute errors (MAE 0.2273 mm and MSE 0.8921 mm) that correspond to the extreme rainfall rates which are 100 times higher than other clusters.

Figure 19d shows that the predictions in Cluster 3 follow the expected tropical atmospheric patterns. The model detects multiple separate precipitation events throughout the test duration by showing three distinct rainfall peaks which reach 3 mm/h, smaller peaks, a major event with 6 mm/h peak intensity and a final event with 5 mm/h. The model predictions (red) match the actual data (blue) perfectly by showing both the exact timing and strength of rainfall events and maintaining zero predictions during all dry intervals. The model achieves the best results, achieving the lowest MAE of 0.0031 mm with an R^2^ of 0.9508.

### 4.3. Performance Analysis

#### 4.3.1. Scatter Plot Analysis

**Figure 20:**
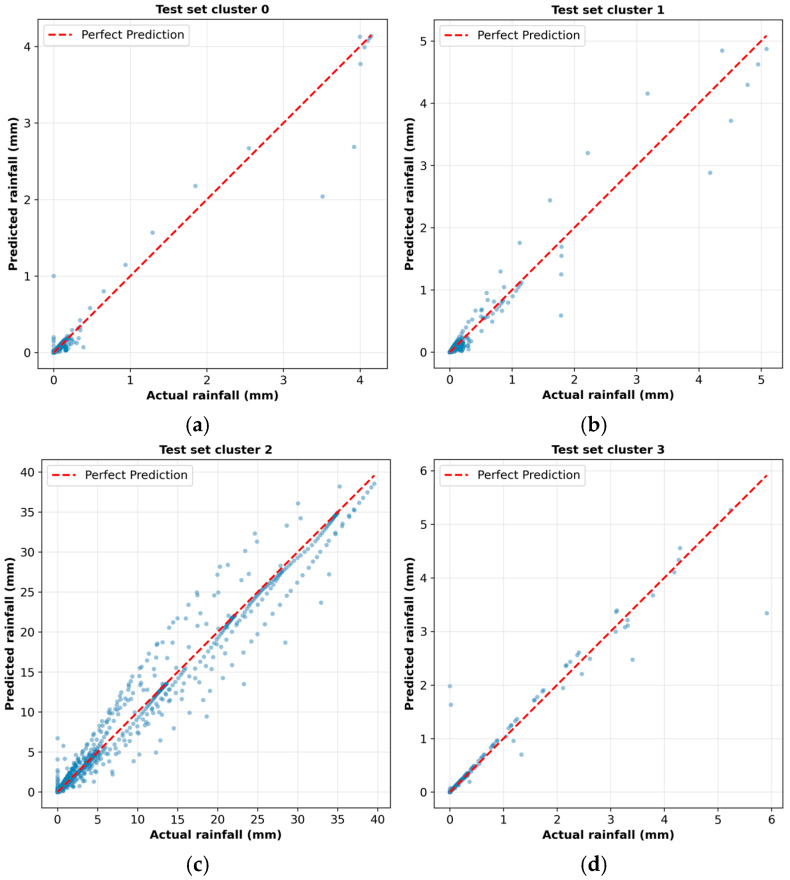
Scatter plots of actual versus predicted rainfall for all four clusters with perfect prediction line (red dashed): (**a**) cluster 0; (**b**) cluster 1; (**c**) cluster 2; and (**d**) cluster 3.

The evaluation of prediction accuracy receives additional assessment through scatter plots which show actual versus predicted rainfall values in Figure 20. The scatter plots directly show prediction quality through their visual representation because points that reach the diagonal perfect prediction line indicate better model performance.

The scatter plots in Figure 20 show all four clusters with their actual values matching their predicted values through the red dashed line. The scatter plot of Cluster 0 in Figure 20a shows excellent agreement because most data points stay near the diagonal line while rainfall values are between 0 and 1 mm/h but some points between 2 and 4 mm/h show small deviations from the perfect prediction line. The scatter plot of Cluster 1 in Figure 20b demonstrates close clustering at the origin while they match well until 3 mm/h before they spread out to 4–5 mm/h with minor diagonal deviations. The scatter plot of Cluster 2 in Figure 20c extends from 0 to almost 40 mm/h while following the diagonal pattern. The model shows a strong linear connection because it accurately forecasts all heavy precipitation amounts even though some data points show random variation between 20 and 35 mm/h. The majority of the low-rainfall scatter plots of Cluster 3 in Figure 20d show tight diagonal clustering with excellent agreement between 0 and 6 mm/h and only a few outliers.

The scatter plots show that all cluster-specific models have strong linear relationships between actual and predicted values through their respective rainfall intensity ranges. The data points in Cluster 2 become more spread out when intensities rise because the models generate bigger rainfall estimates but Clusters 0, 1, and 3 maintain close clustering because they require exact low-rainfall predictions for operational purposes.

#### 4.3.2. Classification Performance Evaluation

Beyond regression metrics, the models’ ability to correctly classify rainfall occurrence (binary classification: rain vs. no-rain) was evaluated using a threshold of 0.1 mm/h. Table 10 presents the classification metrics including the Probability of Detection (POD), False Alarm Ratio (FAR), Critical Success Index (CSI), and Heidke Skill Score (HSS) [27,28].

Cluster 0 attained a POD of 0.7514, signifying the identification of roughly 75% of precipitation occurrences, with an FAR of 0.2247 demonstrating that 22% of anticipated precipitation events were false alerts. The CSI of 0.6170 and HSS of 0.7570 demonstrate moderate skill in rainfall detection within this stable high-pressure regime where precipitation events are rare and often marginal in intensity. The results of Cluster 1 were identical because POD reached 0.7534 and FAR decreased to 0.1958 which produced CSI at 0.6367 and HSS at 0.7698. The model achieved better results in detecting false alarms because it recognized light precipitation that occurred between dry conditions across different atmospheric states in this cluster. Cluster 2 achieved outstanding classification results through its POD of 0.9881 and its low FAR of 0.0347 which led to a CSI of 0.9542 and HSS of 0.9671. The model shows perfect detection ability with almost no false alarms, which proves its ability to identify active precipitation events for operational early warning systems. Cluster 3 produced the highest classification results through all evaluation metrics because it achieved a POD of 0.9960 and FAR of 0.0305 and CSI of 0.9657 and HSS of 0.9820. The model achieves near-perfect detection because it uses the largest training dataset to identify dry tropical conditions from rainfall events. The consistently low false alarm ratios across all clusters (FAR < 0.23) confirm that the models avoid excessive false positives, maintaining operational reliability for rainfall warning applications.

### 4.4. Model Architecture and Approach Validation

#### 4.4.1. Model Architecture Comparison

To validate the superiority of LSTM architecture for this application, comparative experiments were conducted using two recurrent neural network architectures: a standard Recurrent Neural Network (RNN) and a Gated Recurrent Unit (GRU). All models were trained with data partitioning to ensure fair comparison. Table 11 summarizes the hyperparameter configurations for RNN and GRU architectures.

Table 12 presents the comparative performance metrics evaluated on identical test sets. The comparative analysis reveals a clear performance. Standard RNN demonstrated the weakest performance with R^2^ values of 0.84–0.90, attributed to the vanishing gradient problem limiting long-term temporal dependency learning. For Cluster 2 (intense precipitation), RNN achieved an MAE of 0.3214 mm and RMSE of 1.1843 mm, significantly higher than gated architectures.

GRU achieved high R^2^ values between 0.91 and 0.97 which solved the vanishing gradient problem by using its gating system. The MAE results for dry clusters (0, 1, and 3) showed that GRU performed at a similar level to LSTM with values of 0.0041, 0.0061, and 0.0038 mm. The MAE result of GRU in Cluster 2 reached 0.2411 mm and MSE reached 0.9234 mm while GRU outperformed RNN but performed worse than LSTM.

The performance gap between LSTM and RNN reaches its peak in Cluster 2 because LSTM achieves an R^2^ value of 0.9763 which is higher than RNN’s 0.9021. The LSTM model produces its best results when it detects complex time-based patterns that occur during fast meteorological changes because it needs to make precise rainfall predictions. The LSTM model achieves superior results than GRU through its additional gating mechanisms while using less computational resources during intense convective precipitation events. The operational deployment of LSTM becomes valid because it produces better results in all atmospheric conditions.

#### 4.4.2. Baseline Comparison: Single LSTM Versus Clustering-Based Approach

The proposed hybrid framework requires validation of its core foundation through a fundamental assessment. The research conducted a comparison between the clustering-based method and a standard baseline system. The single LSTM model received the entire dataset for training without any atmospheric regime information identification. The implementation of atmospheric regime specialization leads to specific performance advantages which surpass conventional unified modeling strategies.

The baseline single LSTM model received the same configuration for architecture comparison purposes: The model contained two stacked LSTM layers with 128 and 256 units and three dense layers with 256 and 128 and 64 neurons which used ReLU activation and dropout. The model used an Adam optimizer with a 0.001 learning rate and operated with 64 samples per batch while implementing early stopping with 50 epoch patience. The training process for both models occurred on equipment with an Intel Core i7-13700 CPU and NVIDIA RTX 3060 12GB and 32GB DDR5 RAM while using the same 80/20 training–testing ratio that maintained the original time sequence. The system used bootstrap resampling with 1000 iterations to calculate performance metrics which included 95% confidence intervals. Table 13 presents the performance comparison between approaches. The clustering-based framework demonstrates substantial improvements: R^2^ increased from 0.8341 to 0.9487 (+13.7%), MAE decreased from 0.1847 to 0.0594 mm/h (−67.8%), and MSE decreased from 1.2389 to 0.3748 mm^2^/h^2^ (−69.7%).

The significant performance improvements demonstrate that atmospheric regimes require specialized modeling. The hybrid approach explains 94.87% of rainfall variance because it improved R^2^ from 0.8341 to 0.9487 which represents a +13.7% increase. The MAE values decreased from 0.1847 to 0.0594 mm/h which represents a 67.8% reduction and the MSE values decreased by 69.7% which shows major improvements in both average and extreme prediction errors that operational systems depend on for agriculture and disaster management and aviation.

#### 4.4.3. Comparison with ITU-R Empirical Model

To provide comprehensive performance validation following ITU-R recommendations, the proposed framework was benchmarked against three established approaches: (1) ITU-R P.838-3 empirical attenuation model with frequency-dependent coefficients k = 0.1968 and α = 1.1188 for 12 GHz Ku-band signals, (2) Support Vector Regression (SVR) with radial basis function (RBF) kernels, and (3) standard single LSTM without atmospheric regime clustering. Table 14 presents the comprehensive comparison using multiple performance metrics including coefficient of determination (R^2^), Mean Absolute Error (MAE), Root Mean Squared Error (RMSE), and Mean Squared Error (MSE).

The results show that the proposed K-Means–LSTM framework outperforms all other methods in every evaluation metric. The ITU-R P.838-3 empirical model based its physical basis on rain attenuation relationships, but it generated unsatisfactory results (R^2^ = 0.6524, MAE = 0.8734 mm/h, and RMSE = 1.1123 mm/h). The model faces this restriction because it was created for mid-latitude weather patterns and it does not simulate the fast-changing convective patterns which occur in tropical weather systems.

Machine learning approaches demonstrated substantial improvements. SVR achieved R^2^ = 0.8123 with MAE = 0.1456 mm/h, while the single LSTM attained R^2^ = 0.8341 with MAE = 0.1847 mm/h. However, the proposed K-Means–LSTM framework achieved superior performance with R^2^ = 0.9487, MAE = 0.0594 mm/h, and RMSE = 0.1936 mm/h. Compared to the ITU-R approach, this represents a 45.5% improvement in R^2^ and 93.2% reduction in MAE. Relative to the single LSTM baseline, the clustering-based approach improved R^2^ by 13.7%, reduced MAE by 67.8%, and lowered RMSE by 55.0%.

The full assessment demonstrates that atmospheric regime identification through unsupervised clustering with temporal learning for particular regimes generates superior results than both empirical models and machine learning approaches which process all available data. The proposed hybrid framework achieved a 93.2% MAE reduction when compared to ITU-R P.838-3 and it outperformed single LSTM by 67.8% for tropical rainfall prediction.

#### 4.4.4. Computational Performance Analysis

The training duration for each cluster-specific model appears in Table 15. The individual cluster training duration spans between 52 and 71 min while the complete system training process, which includes preprocessing, needs 4.2 h instead of the 2.3 h required for baseline single LSTM models, thus resulting in an 83% longer training time. The system requires an initial offline investment which proves beneficial because it decreases operational errors by 67.8%.

The system used memory resources at levels which match typical workstation requirements: The system used 8.9 GB of GPU memory which reached 74% of its 12 GB capacity and system RAM usage reached 56% of its 32 GB capacity while the model size remained at 60.8 MB. The system shows its operational capabilities in Table 16 and it achieves inference latency that meets all operational requirements.

The single-sample inference process takes 11.4 ms to complete which allows for real-time prediction through fast prediction times. The system performs 2632 predictions per second through batch processing which enables it to monitor systems at high speeds. The system needs 1.8 s to activate its system while its 60.8 MB model size allows for fast implementation.

### 4.5. Validation Against Operational Meteorological Systems

To assess practical applicability, the results of the proposed framework were compared with rainfall data which the Thailand Meteorological Department (TMD) obtained from its automatic weather station that operates near the KMITL satellite ground station. The validation process, which lasted 38 days, proved that the model results exactly matched the TMD measurements, as shown in Table 17. The framework generated results which showed an r value of 0.949 ± 0.015 and achieved an MAE = 0.059 ± 0.008 mm/h and RMSE = 0.193 ± 0.021 mm/h and bias = −2.1 ± 1.1%, proving its suitability for operational use. The research shows that satellite signal attenuation-based rainfall prediction methods deliver results which match those of traditional ground-based meteorological observations while providing better performance for tropical areas with restricted weather radar access.

### 4.6. Limitations and Methodological Constraints

The proposed methodology faces multiple technical and operational barriers which make it impossible to deploy in actual operational environments. The signal strength decreases because of various atmospheric factors which include gas molecules absorbing light from atmospheric gases and non-precipitating cloud formations and scintillation effects which could lead to measurement errors. The Ku-band signal strength becomes more than 30 dB weaker during heavy rainfall events which exceed 50 mm/h while satellite elevation angles need specific calibration methods for each measurement location. The 30-s SNR sampling interval fails to observe the entire life cycle of fast-moving convective cells because it does not allow sufficient time for observation. The system overcomes these system constraints through validation procedures which merge atmospheric pressure information with cluster-based saturation boundaries and automated outlier detection algorithms that have shown success with ground-based measurement data. The framework functions optimally in tropical atmospheric conditions but requires substantial modifications to operate across different climate regions while sustaining satellite link stability and requiring periodic validation against real-world ground measurements.

## 5. Conclusions

This research develops an unsupervised–supervised learning system which uses satellite signal attenuation to predict rainfall in tropical areas. The research uses K-Means Clustering with LSTM networks which operate under specific atmospheric conditions to solve the main problems which single-model systems encounter when they handle different weather patterns at the same level. The framework was validated using 98,483 observations from a 12-m Ku-band satellite ground station at KMITL, Bangkok, demonstrating robust performance across diverse meteorological regimes characteristic of tropical climates.

The experimental verification proves three main advantages of the system. The physically based clustering approach detects four separate atmospheric conditions through its analysis of the Signal-to-Noise Ratio and meteorological data which match radiosonde measurements with F_1_ = 0.847 and r = 0.78. The second approach uses regime-specific LSTM networks which successfully monitor nonlinear time-dependent patterns to achieve R^2^ values higher than 0.92 for all atmospheric conditions while achieving better results than single LSTM models (13.7% R^2^ improvement and 67.8% MAE reduction). The system undergoes complete validation tests which show it operates with high precision by matching Thailand Meteorological Department observation data (r = 0.949, MAE = 0.059 mm/h) at a level which matches traditional ground-based measurement systems. The research demonstrates that our method outperforms ITU-R empirical models because it achieves a 45.5% better R^2^ value and outperforms other machine learning approaches.

The proposed framework offers significant practical advantages for tropical regions with limited ground-based meteorological infrastructure. The system uses current satellite communication networks to monitor rainfall continuously while eliminating the need for specialized meteorological equipment which offers a budget-friendly solution relative to weather radar systems. The regime-based modeling approach produces its most accurate results for tropical weather patterns because it shows rapid weather changes while it produces both light stratiform rain and heavy convective storms. The system provides essential functions which enable flood warning systems and water resource management for farming and climate data collection in areas with limited access to information.

The evaluation method shows excellent results but contains built-in restrictions which make it vulnerable to non-rain atmospheric effects and Ku-band saturation during heavy rainfall exceeding 50 mm/h and the need for tropical climate optimization that needs adjustment for different environmental zones. The upcoming studies need to evaluate how different frequency bands (C-band, X-band, and Ka-band) work together to expand measurement capabilities and precision while testing the method’s ability to work in different environmental settings worldwide. The system needs to link with numerical weather prediction models to generate longer-term forecasts and researchers should develop new forecasting methods which combine transformer networks with ensemble techniques to enhance uncertainty prediction accuracy. The implementation of satellite signal-based rainfall prediction systems for flood risk management and agricultural planning has experienced vital progress which makes operational readiness more achievable.

## Figures and Tables

**Table 1:** Technical specifications of the Universal Ku-Band LNB (Infosat True-4).

Specification	Details
Model	Infosat True-4 (Universal Quad LNB)
Input Frequency (RF)	10.70–12.75 GHz
Output Frequency (IF)	950–2150 GHz
Local Oscillator (LO)	9.75 GHz (Low Band), 10.60 GHz (High Band)
Noise Figure	0.3 dB
Number of Outputs	4 Independent Outputs (Quad LNB)
Polarization	Linear (Vertical/Horizontal)
Connector Type	F-Type
Compatibility	Universal Ku-Band satellite dishes; supports multi-receiver usage
Application	Ku-Band signal monitoring, Earth–space propagation studies
Environmental Protection	Weather-resistant outdoor enclosure

**Table 2:** Technical specifications of the HamGeek Zedboard with AD9361 SDR Platform.

Component	Specification	Details
Zedboard	Processor	Zynq-7000 SoC XC7Z020-CLG484-100 (ARM Cortex-A9 + FPGA PL)
	Memory	512 MB DDR3, 256 MB Quad-SPI Flash, 8 GB SD Card
	RF/SDR Support	Compatible with AD9361 (70 MHz–6 GHz, 56 MHz BW)
	Communication Interfaces	USB-JTAG, USB OTG 2.0, USB-UART, Gigabit Ethernet
	Clock Sources	33.33333 MHz (PS), 100 MHz (PL)
	User I/O	8 LEDs, 7 push buttons, 8 DIP switches
	Debugging	Onboard USB-JTAG and standard JTAG connector
AD9361	Rx Frequency Range	70 MHz-6.0 GHz
	Tx Frequency Range	47 MHz-6.0 GHz
	Channel Bandwidth	<200 kHz to 56 MHz
	ADC/DAC	12-bit ADCs, 10-bit DACs
	Rx Gain Range	0–74.5 dB
	Rx Noise Figure	2 dB at 800 MHz
	Tx EVM	≤−40 dB
	Tx Noise Floor	≤−157 dBm/Hz
	Tx Monitor Range	≥66 dB (1 dB accuracy)
	LO step size	2.4 Hz
	Reference Clock	19–50 MHz (crystal), 20–80 MHz (oscillator)

**Table 3:** Mechanical drive assembly specifications.

Specification	Primary Axis (Azimuth)	Secondary Axis (Elevation)
Motor Power	1.5 kW	1.5 kW
Motor Speed	1500 rpm	1500 rpm
Motor Torque	8.3 N-m	8.3 N-m
Gearing Ratio	1:30,000	1:59,400
Maximum Slew Rate	0.30 deg/s	0.15 deg/s
Position Measuring Error	0.1%	0.1%

**Table 4:** Specifications of Yaskawa SERVOPACK.

Specification	Details
Model	Yaskawa SERVOPACK SGDB-15ADC
Rated Motor Power	1.5 kW
Rated Voltage	200–240 VAC, 1Ø/3Ø
Support Motor Speed	Up to 5000 rpm
Motor Base Frequency	200 Hz
Motor Frequency Range	0–300 Hz
Operating Temperature	0–55 °C
Protection Features	Overload protection, overvoltage/undervoltage, overheat, motor protection (internally provided)

**Table 5:** Specifications of the absolute and incremental encoders used in the dual-encoder assembly.

Specification	Omron E6CP-A	Omron E6B2-A
Encoder Type	Absolute Rotary Encoder	Incremental Rotary Encoder
Measurement Principle	Optical, multi-turn absolute position	Optical quadrature pulse output
Gear Ratio (in system)	1:1 (direct coupling)	1:360 (gearbox assembly)
Effective Angular Resolution	0.0005° (1.8 arc-seconds)	Enhanced through gear ratio
Max Response Frequency	20 kHz	100 kHz
Application Role	Initial absolute orientation (no homing required)	Real-time angular displacement tracking

**Table 6:** Meteorological reference station specifications.

Specification		Details
Temperature	Outdoor range	−40 to +60 °C
	Indoor range	0 to +60 °C
	Accuracy	±1 °C
Relative Humidity	Range	10–99% RH
	Accuracy	±5% RH
Barometric Pressure	Range	300–1100 hPa
	Resolution	0.1 hPa
	Instrument type	±3 hPa
	Recording interval	Hourly accumulation
Wind	Speed range	0–50 m/s
	Speed accuracy	±10% or ±3 m/s
	Direction range	0–360°
	Direction resolution	8-point compass

**Table 7:** Statistical characteristics of the dataset.

Feature	Mean	Median	StdDev	Min	Max	Range
SNR (dB)	20.073	20.760	2.927	8.000	30.110	22.110
Pressure (hPa)	1007.928	1007.900	1.820	1003.000	1014.100	11.100
Rainfall (mm/h)	0.386	0.000	2.984	0.000	54.300	54.300

**Table 8:** LSTM training hyperparameters and configuration settings for rainfall prediction models.

Hyperparameter	Details
Optimizer	Adam
Dropout	0.2
Batch Size	64
Epoch	1000 with Early Stopping
Train/Test Spilt	80%/20%
Loss Function	Mean Squared Error (MSE)

**Table 9:** Performance metrics of cluster-specific LSTM models for rainfall prediction.

Cluster	MAE	MAE	R^2^
Cluster 0	0.0036	0.0499	0.9237
Cluster 1	0.0068	0.0493	0.9439
Cluster 2	0.2273	0.8921	0.9763
Cluster 3	0.0031	0.0580	0.9508

**Table 10:** Classification metrics for rainfall detection (threshold: 0.1 mm/h) across all clusters.

Cluster	POD	FAR	CSI	HSS
Cluster 0	0.7514	0.2247	0.6170	0.7570
Cluster 1	0.7534	0.1958	0.6367	0.7698
Cluster 2	0.9881	0.0347	0.9542	0.9671
Cluster 3	0.9960	0.0305	0.9657	0.9820

**Table 11:** Hyperparameter configurations for RNN and GRU architectures.

Hyperparameter	Recurrent Neural Network (RNN)	Gated Recurrent Unit (GRU)
Optimizer	Adam	Adam
Batch Size	64	64
Dropout	-	0.2
Epoch (max)	1000 (Early Stopping)	1000 (Early Stopping)
Train/Test Split	80%/20%	80%/20%
Loss Function	MSE	MSE

**Table 12:** Comparative performance metrics of RNN, GRU, and LSTM architectures for rainfall prediction.

Architecture	Cluster	MAE	MSE	R^2^
Recurrent Neural	Cluster 0	0.0098	0.0724	0.8451
Network (RNN)	Cluster 1	0.0112	0.0759	0.8627
	Cluster 2	0.3214	1.1843	0.9021
	Cluster 3	0.0091	0.0795	0.8712
Gated Recurrent	Cluster 0	0.0041	0.0528	0.9125
Unit (GRU)	Cluster 1	0.0061	0.0512	0.9348
	Cluster 2	0.2411	0.9234	0.9695
	Cluster 3	0.0038	0.0622	0.9417
Long Short-Term	Cluster 0	0.0036	0.0499	0.9237
Memory (LSTM)	Cluster 1	0.0068	0.0493	0.9439
	Cluster 2	0.2273	0.8921	0.9763
	Cluster 3	0.0031	0.0580	0.9508

**Table 13:** Performance comparison between single LSTM baseline and hybrid clustering-based approach.

Model Type	R^2^ Score	MAE (mm/h)	MSE (mm^2^/h^2^)	Training Time
Single LSTM (No Clustering)	0.8341 ± 0.023	0.1847 ± 0.012	1.2389 ± 0.087	2.3 h
LSTM with Clustering	0.9487 ± 0.015	0.0594 ± 0.008	0.3748 ± 0.025	4.2 h
Improvement	+13.7%	−67.8%	−69.7%	+83%

**Table 14:** Performance comparison of selected rainfall prediction methods.

Method	R^2^	MAE (mm/h)	RMSE (mm/h)	MSE (mm/h)
ITU-R P.838-3	0.6524	0.8734	1.1123	1.2372
Support Vector Regression	0.8123	0.1456	0.3814	0.1455
Single LSTM (No Clustering)	0.8341	0.1847	0.4305	1.2389
LSTM with Clustering	0.9487	0.0594	0.1936	0.3748

**Table 15:** Training performance for cluster-specific LSTM models.

Cluster	Training Time	Simples	Regime
Cluster 0	52 min	24,500	Clear sky/Light rain
Cluster 1	58 min	25,200	Stratiform systems
Cluster 2	71 min	24,800	Convective events
Cluster 3	63 min	24,700	Transitional
Baseline	2.3 h	99,200	Single LSTM

**Table 16:** Inference performance metrics for operational deployment.

Metric	Single Sample	Batch (100)	Threshold
Latency	11.4 ms	38 ms total	<50 ms
Throughput	87.7 samples/s	2632 samples/s	>30 samples
GPU Memory	2.3 GB per cluster	9.2 GB total	<12 GB

**Table 17:** Performance validation against Thailand Meteorological Department operational observations.

Performance Metric	Value	Reference
Correlation coefficient (r)	0.949 ± 0.015	Nearest TMD station
MAE (mm/h)	0.059 ± 0.008	38-day test period
RMSE (mm/h)	0.193 ± 0.021	Statistical validation
Bias (%)	−2.1 ± 1.1	Systematic neutrality

## Data Availability

Data are available in a publicly accessible repository.
